# ASSESSING HEALTH-RELATED OUTCOMES OF MEDICAL CANNABIS USE AMONG OLDER PERSONS IN COLORADO AND ILLINOIS

**DOI:** 10.1093/geroni/igz038.738

**Published:** 2019-11-08

**Authors:** J Alton Croker, Julie Bobitt, Kanika Arora, Brian Kaskie

**Affiliations:** 1 University of Iowa, Iowa City, Iowa, United States; 2 UNIVERSITY OF ILLINOIS AT URBANA-CHAMPAIGN, Champaign, Illinois, United States

## Abstract

The use of cannabis is increasing among older Americans. A growing body of evidence shows cannabis and cannabinoids effective for treating chronic pain, spasticity, nausea, and sleep disturbances. States seeking to respond to the treatment needs of specific patient populations have legalized the use of cannabis for medical purposes. Few instruments offer standard outcomes for understanding the use of medial cannabis from the patient perspective, particularly focusing on older persons. Using cross-sectional survey data from a sample of older persons in Colorado and Illinois, we validate two scales to consistently measure patient-reported health related outcomes of medical cannabis use. We confirmed the validity of two separate, reliable outcome scales: a three-factor scale for measuring global health outcomes and a single-factor scale for capturing adverse health events. The COPS questionnaire revealed strong construct validity and internal consistency, and a lack of meaningful factor variance.

